# Supplementary material for: Causal interplay between lactose intolerance and gut microbiota: a combined bidirectional Mendelian randomization and in vivo validation study
Source: Front Nutr. 2026 Jun 1;13:1803337. doi: 10.3389/fnut.2026.1803337 (PMC13265576; doi:10.3389/fnut.2026.1803337)
Supplement: Supplementary file 1 [file Data_Sheet_1.zip › supplementary materials/Forward/leave one out cross validation/ebi-a-GCST90027604.finngen_R12_E4_LACTONAS.pdf]

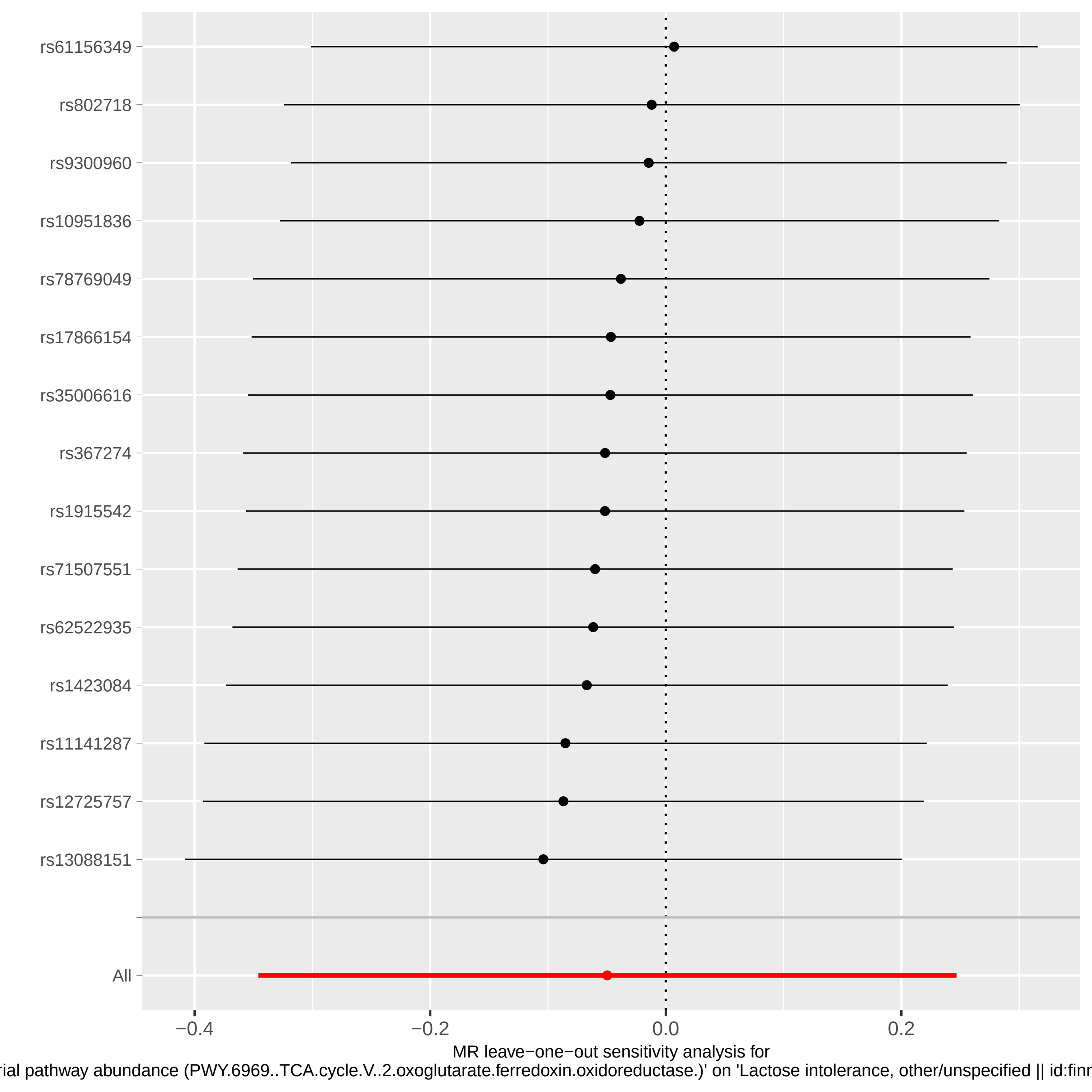
rs61156349
rs802718
rs9300960
rs10951836
rs78769049
rs17866154
rs35006616
rs367274
rs1915542
rs71507551
rs62522935
rs1423084
rs11141287
rs12725757
rs13088151
All
−0.4
−0.2
0.0
0.2
MR leave−one−out sensitivity analysis for
rial pathway abundance (PWY.6969..TCA.cycle.V..2.oxoglutarate.ferredoxin.oxidoreductase.)' on 'Lactose intolerance, other/unspecified || id:finn
